# Quinine

**Published:** 1887-04

**Authors:** 


### QUININE.

The famous chinchona tree of Peru, from the bark whereof the quinine of commerce is extracted, derived its name from the Countess of Chinchona, wife of the Viceroy of Peru, who was cured by it of a tertian ague, in 1638. On the return of the Count and Countess to Spain they took with them a quantity of this healing bark, and were the first to introduce it as a medicine to the "old world," where it was known for some time as Countess bark or powder. The tree was subsequently named by Linneæus, the celebrated naturalist, in recognition of the great service the Countess had rendered to mankind.

In 1670, some Jesuit missionaries sent parcels of the powdered bark to Rome, whence it was distributed throughout Europe by Cardinal du Lugo, and employed in the cure of agues with great success. From this circumstance it was often called Jesuit's bark and Cardinal's bark, on account of which its use was, for a time, rejected by the Protestants, and even physicians opposed its employment as dangerous to health. The native Peruvians called it *yunyuena*, which signifies bark of barks, and its virtues were known to them long before the Spanish invasion.

Forests of chinchona trees are found throughout the more northerly region of the Cordilleras. When exploring new districts, it is the custom for the Carilleros, or bark collectors, to make their way into the forest, accompanied by an experienced searcher, called the Coleador, who, upon climbing one of the taller forest trees, is able to distinguish groups of chinchonas by their dark foliage and the peculiar light reflected from their leaves. Upon reaching a favorable spot, the party erect temporary huts and commence operations in earnest. The trees usually attain a height of from forty to sixty feet. They first fell a tree, cutting near the ground, to stimulate the growth of other trees from new shoots. The bark is then stripped off and dried, care being observed to keep it dry and free from mold. When sufficiently cured, it is made into bundles of a convenient size for packing, and is often carried for long distances on the backs of native Peruvians.

There are several varieties of chinchona trees, but the only ones found to be available for medicinal purposes are the red and the grey bark, the red being the more valuable of the two. Some Peruvian families have carried on the business of bark gathering for many generations, and the product of their labors has kept the field as the one essential specific for agues and other malarial diseases for upwards of two centuries.

